# The Invisible Caseload in Plain Sight: Psychiatric Presentation at Dermatology's Front Door- a Call to Action for Psychiatrists.

**DOI:** 10.1192/bjo.2026.11610

**Published:** 2026-06-30

**Authors:** Augusta Okoro

**Affiliations:** St Andrew's Healthcare, Northampton, United Kingdom

## Abstract

**Aims:**

Skin disease carries a high burden of psychological morbidity and dermatology often functions as the first clinical contact for primary psychiatric presentations.

We aim to:

* Quantify psychiatric morbidity presenting to dermatology.

* Assess progress since the All-Party Parliamentary Group on Skin (APPG) report on Mental Health and Skin Disease (2020).

* Map psychiatry’s engagement in psycho-dermatology across curricula, training, and research.

* Evaluate practical Royal College of Psychiatrists (RCPsych) and British Association of Dermatologists (BAD) solutions for service development.

**Methods:**

Exploratory mapping of authoritative UK sources to characterise clinical need, service models, training, and research activity. Reviewed materials included the APPG (2020) report, BAD and Psychodermatology UK resources, UK service evaluations, RCPsych curricula and liaison guidance, and recent publication-trend analyses. Emphasis was placed on high-impact findings and actionable service metrics.

**Results:**

Evidence indicates a substantial psychiatric caseload presents to dermatology and is under-recognised by mainstream psychiatric services:

**1. Burden:** The APPG survey reports 98% of patients experience negative psychological impact, with 5% offering spontaneous disclosures of suicidal ideation, evidencing substantial unmet psychiatric morbidity.

**2. First Contact:** Dermatologists frequently receive first presentations of DI, BDD, and complex psychological distress. They explicitly highlight the need for psychiatric presence given limited psychiatric training.

**3. Service and Commissioning gaps**: Integrated pilots show benefit from liaison psychiatry, yet provision remains patchy. Dermatology stakeholders note that complex psycho-dermatology consultations align more closely with psychiatry tariffs, underscoring commissioning gaps.

**4. Curriculum gap and Competency mapping**: The Royal College encourages special Interest Development with provision of 2 session weekly. However, recent RCPsych Liaison Curriculum contains minimal psychodermatology-specific content or competencies, and pathways to competency are not clearly defined.

**5. Research gap:** The UK research base on this topic remains predominantly dermatology-led, with psychiatry perspectives under-represented. The APPG report calls for multidisciplinary research networks and collaboration.

**6. Training outside psychiatry:** Structured psychodermatology education is developing via BAD/Psychodermatology UK but sits outside RCPsych governance. There is no clear endorsement of these pathways from the RCPsych.

**Conclusion:**

Dermatology clinics host a psychiatric caseload that psychiatry seldom encounters, contributing to missed diagnoses and unmet risk needs. The explicit calls from dermatologists for psychological/psychiatric involvement and tariff alignment, highlights clear structural gaps. The evidence supports RCPsych-endorsed optional psycho-dermatology pathways, BAD-linked clinical rotations, development of College guidance, and Psycho-dermatology special interest groups. These targeted steps would bring this invisible caseload into psychiatry's view and strengthen diagnostic accuracy, patient safety, and multidisciplinary mind–skin care.

